# A Comprehensive Review on High-Flow Nasal Cannula Oxygen Therapy in Critical Care: Evidence-Based Insights and Future Directions

**DOI:** 10.7759/cureus.66264

**Published:** 2024-08-06

**Authors:** Shubham Petkar, Dnyanshree Wanjari, Vishnu Priya

**Affiliations:** 1 Anaesthesiology, Jawaharlal Nehru Medical College, Datta Meghe Institute of Higher Education and Research, Wardha, IND

**Keywords:** humidified oxygen therapy, acute respiratory failure, non-invasive ventilation, respiratory support, critical care, high-flow nasal cannula (hfnc)

## Abstract

High-flow nasal cannula (HFNC) therapy has emerged as a significant advancement in respiratory support, offering a non-invasive alternative to traditional oxygen delivery methods in critical care settings. This review comprehensively evaluates HFNC therapy, focusing on its definition, historical evolution, and current clinical applications. HFNC therapy delivers humidified and heated oxygen at high flow rates through a nasal cannula, enhancing oxygenation and patient comfort. The review highlights the physiological mechanisms underlying HFNC and its efficacy in managing acute respiratory failure, chronic obstructive pulmonary disease exacerbations, and postoperative respiratory support. Key findings from clinical trials and meta-analyses are discussed, emphasizing HFNC's advantages over conventional methods, such as reduced intubation rates and shorter ICU stays. The review also addresses safety considerations, including potential risks and complications associated with HFNC therapy. Furthermore, it explores future directions for research and technological advancements aimed at optimizing HFNC use in diverse patient populations. This review aims to provide evidence-based insights to inform clinical practice and guide future investigations in respiratory therapy.

## Introduction and background

High-flow nasal cannula (HFNC) therapy is a form of respiratory support that delivers a high flow of humidified and heated oxygen through a nasal cannula. Unlike traditional oxygen therapy, which uses lower flow rates and non-humidified gases, HFNC provides a continuous air stream at flow rates exceeding 60 liters per minute [1]. This therapy enhances oxygenation and improves respiratory function in patients with acute and chronic respiratory failure. HFNC therapy is distinguished by its ability to deliver precise oxygen concentrations, maintain airway patency, and reduce the work of breathing, making it a valuable tool in managing critically ill patients [2]. The concept of high-flow nasal oxygen therapy has evolved over several decades, driven by advancements in respiratory technology and a deeper understanding of respiratory physiology. Initially, oxygen was administered through low-flow nasal cannulas or masks, which often led to discomfort and inadequate gas exchange [3]. The development of HFNC technology in the early 2000s represented a significant shift, leveraging heated and humidified air to improve patient comfort and therapeutic efficacy. Early clinical studies demonstrated the potential benefits of HFNC over conventional methods, leading to broader adoption in critical care settings. Since its introduction, HFNC therapy has continuously refined, with ongoing research exploring its applications and optimizing its use in various patient populations [3].

In critical care settings, HFNC therapy plays a crucial role in managing patients with acute respiratory distress, hypoxemic and hypercapnic respiratory failure, and other severe respiratory conditions. Its ability to provide high-flow, humidified oxygen makes it an effective alternative to more invasive interventions, such as mechanical ventilation, and a valuable option for patients who may not tolerate or require invasive support [4]. HFNC therapy has improved patient outcomes, including reduced intubation rates, shorter ICU stays, and enhanced patient comfort. Its non-invasive nature also makes it a preferred choice for early respiratory support and a potential bridge to more intensive treatments if necessary [4]. This review aims to provide a comprehensive analysis of HFNC therapy in the context of critical care. By examining the current evidence on its efficacy, safety, and clinical applications, this review seeks to offer insights into the role of HFNC in modern respiratory management. It will explore the mechanisms underlying HFNC therapy, evaluate its performance compared to traditional oxygen delivery methods, and identify critical areas for future research. The goal is to equip clinicians and researchers with evidence-based knowledge to optimize the use of HFNC therapy and enhance patient care in critical care settings.

## Review

Mechanism of action

Principles of HFNC Oxygen Delivery

**Figure 1:**
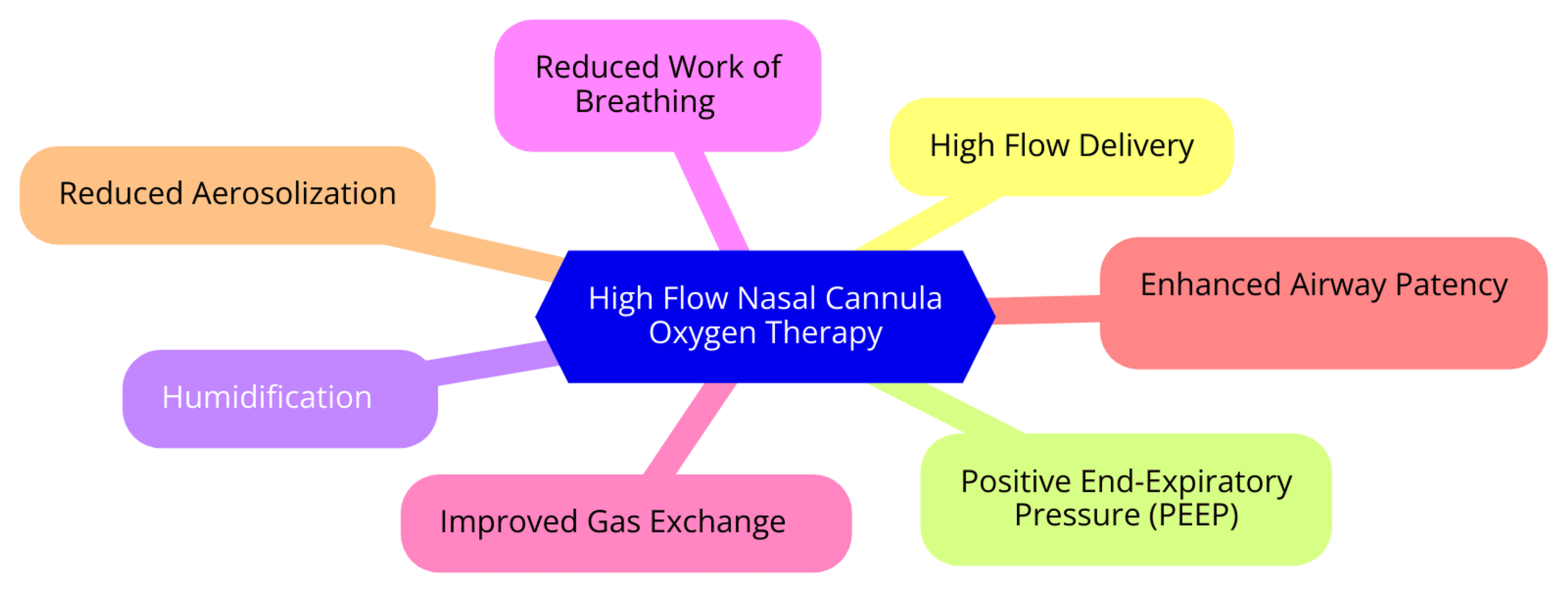
The mechanism of action of high-flow nasal cannula oxygen therapy Image credit: Dr. Shubham Petkar

HFNC oxygen therapy employs several principles to deliver oxygen effectively, particularly to patients in respiratory distress. The HFNC system comprises an air/oxygen blender, an active heated humidifier, a single heated circuit, and a wide-bore nasal cannula. This configuration enables the delivery of heated, humidified oxygen at high flow rates, reaching up to 60 L/min [5]. A primary mechanism of HFNC is precise oxygen delivery. HFNC provides a significantly higher flow rate than traditional nasal cannulas, which typically deliver 2-6 L/min. This high flow rate meets or exceeds the patient’s peak inspiratory flow demands, minimizing the entrainment of room air and ensuring the prescribed oxygen concentration reaches the lungs effectively [5]. HFNC also enhances functional residual capacity (FRC) by creating a modest positive pressure effect, crucial for improving oxygenation, particularly in patients with atelectasis or reduced lung volumes. Studies indicate that FRC can increase by approximately 25% when using HFNC [6]. Another vital mechanism is dead space washout. The continuous high oxygen flow helps to wash out the anatomical dead space in the upper airways and proximal tracheobronchial tree. This washout improves ventilation efficiency by reducing carbon dioxide rebreathing and enhancing overall gas exchange [7]. Furthermore, HFNC reduces the work of breathing by delivering heated and humidified gas at high flow rates. This reduces respiratory distress, as the therapy lessens the effort required to inhale, improving comfort and treatment compliance [2]. The clinical benefits of HFNC include improved patient comfort, decreased need for escalation of care, and versatile application. The warmth and humidity of the inspired air enhance tolerance compared to traditional oxygen delivery methods, leading to better patient compliance [8]. HFNC has been shown to reduce the need for more invasive forms of respiratory support, such as intubation, in patients with mild to moderate hypoxemic respiratory failure. In addition, HFNC is effective in various clinical scenarios, including post-extubation support, peri-intubation oxygenation, and treatment of acute hypoxemic respiratory failure [8]. The mechanism of action of HFNC oxygen therapy is shown in Figure 1.

Physiological Effects on the Respiratory System

HFNO therapy has significant physiological effects on the respiratory system, making it particularly beneficial for patients experiencing acute respiratory failure. One of the primary advantages of HFNO is its ability to improve oxygenation [9]. HFNO maintains a higher fraction of inspired oxygen (FiO_2_) by delivering a high flow of heated and humidified oxygen, effectively reducing oxygen dilution with room air. This leads to improved arterial oxygen levels (PaO_2_) in patients with hypoxemic respiratory failure, crucial for maintaining adequate tissue oxygenation [9]. Another critical physiological effect of HFNO is the reduction in breathing work. Studies have demonstrated that HFNO decreases inspiratory effort and minute ventilation while stabilizing arterial carbon dioxide levels (PaCO_2_) [10]. This reduction in respiratory workload is particularly beneficial for preventing respiratory muscle fatigue, allowing patients to breathe more efficiently during episodes of distress. By promoting a more effective breathing pattern, HFNO aids in recovering patients facing respiratory challenges [10]. HFNO also plays a vital role in increasing end-expiratory lung volume, which helps recruit collapsed alveoli and improve lung compliance. This increase in lung volume enhances ventilation and gas exchange distribution, reducing the risk of atelectasis and promoting overall lung function [11]. Furthermore, the therapy improves ventilation homogeneity, ensuring airflow is evenly distributed throughout the lungs. This effect is especially important for patients with conditions such as chronic obstructive pulmonary disease (COPD) or acute respiratory distress syndrome (ARDS), where uneven ventilation can exacerbate hypoxemia [11]. In addition, HFNO generates a mild positive end-expiratory pressure (PEEP), which helps keep the airways open and prevents alveolar collapse at the end of expiration. This PEEP effect improves lung mechanics and enhances oxygenation and ventilation efficiency. The provision of humidified gas also supports mucociliary clearance, which is essential for maintaining airway patency and preventing infections. By preventing the drying of secretions, HFNO enhances the efficiency of the mucociliary transport system, promoting better respiratory health [12]. Importantly, research indicates that HFNO has minimal impact on hemodynamic parameters and cardiovascular stress biomarkers, making it a safe option for patients with underlying cardiovascular issues. This safety profile, combined with its physiological benefits, underscores the value of HFNO therapy in critical care settings [13].

Comparison With Other Oxygen Delivery Methods

HFNO therapy has gained prominence in critical care settings, particularly for managing respiratory failure. Its effectiveness is often compared to other oxygen delivery methods, such as non-invasive ventilation (NIV) and Venturi masks. Each method has unique mechanisms of action, benefits, and limitations, making it essential for clinicians to understand these differences to optimize patient care [14]. HFNO delivers heated and humidified oxygen at up to 60 L/min flow through a wide-bore nasal cannula. This approach offers several advantages, including improved oxygenation. HFNO can enhance oxygen delivery more effectively than standard low-flow systems, including Venturi masks, for the same fraction of inspired oxygen (FiO_2_) [15]. In addition, patients typically find HFNO more comfortable than NIV, as it is less restrictive and does not involve a tight-fitting mask, which can lead to discomfort and feelings of claustrophobia. Moreover, HFNO reduces respiratory rates and the overall work of breathing, making it particularly beneficial for patients experiencing acute respiratory failure. However, HFNO may not provide the same degree of alveolar recruitment as NIV, which can be a limitation in cases of severe respiratory distress [15]. On the other hand, NIV delivers pressurized, oxygenated gas through a mask, assisting ventilation and oxygenation. One of its key benefits is its ability to recruit collapsed alveoli more effectively than HFNO, which can lead to improved oxygenation, especially in patients with conditions like COPD or acute cardiogenic pulmonary edema (ACPE) [16]. However, NIV is often associated with discomfort, leading to issues with patient compliance. High failure rates have been reported, particularly in patients with acute hypoxemic respiratory failure, which can increase morbidity and mortality in this population [16]. The Venturi mask is another low-flow oxygen delivery device that mixes room air with oxygen to achieve a specific FiO_2_. While it allows for precise control of oxygen delivery, it generally provides less effective oxygenation than HFNO for the same FiO_2_ settings [17]. Studies have shown that HFNO leads to better oxygenation and lower respiratory rates than the Venturi mask. Furthermore, the Venturi mask can be less comfortable, resulting in higher interface displacement and discomfort during use, which may hinder its effectiveness [17].

Clinical applications

Acute Respiratory Failure

Acute respiratory failure is a critical condition requiring immediate medical intervention and can be classified into two primary types: hypoxemic respiratory failure and hypercapnic respiratory failure. Understanding these distinctions is essential for effective management and treatment [18]. Hypoxemic respiratory failure occurs when there is insufficient oxygen in the blood, known as hypoxemia. This type of respiratory failure primarily results from impaired gas exchange in the lungs, which can arise from various underlying conditions. Common causes include pneumonia, ARDS, pulmonary edema, and interstitial lung disease. In these situations, the lungs cannot adequately oxygenate the blood, leading to low oxygen saturation levels [18]. Patients with hypoxemic respiratory failure typically present with symptoms such as rapid breathing, shortness of breath, and cyanosis (a bluish tint to the skin). The primary goal of treatment in these cases is to restore adequate oxygen levels, often through supplemental oxygen therapy or NIV techniques like HFNC or continuous positive airway pressure (CPAP) [18]. By contrast, hypercapnic respiratory failure is characterized by elevated levels of carbon dioxide in the blood, known as hypercapnia. This condition arises when the lungs fail to eliminate carbon dioxide effectively, leading to its accumulation in the bloodstream [19]. Common causes of hypercapnic respiratory failure include central nervous system disorders (such as head injuries or drug overdoses), neuromuscular diseases (like Guillain-Barré syndrome or myasthenia gravis), and severe airway obstruction (as seen in COPD). Patients experiencing hypercapnic respiratory failure may exhibit symptoms such as confusion, lethargy, and headache, along with signs of respiratory distress. Treatment strategies often focus on improving ventilation, including NIV, bilevel-positive airway pressure (BiPAP), or invasive mechanical ventilation (IMV) in more severe cases [19].

COPD Exacerbations

COPD exacerbations are acute episodes characterized by a worsening of respiratory symptoms, leading to increased dyspnea, cough, and sputum production. These exacerbations can significantly impact a patient's quality of life and often necessitate hospitalization. The primary goals in managing COPD exacerbations are to relieve symptoms, restore functional capacity, and prevent further complications [20]. Oxygen therapy is a critical component of treatment for COPD exacerbations. Patients typically require supplemental oxygen to maintain 90% or higher saturation levels. In more severe cases, especially when hypercapnia is present, intubation or positive-pressure ventilation may be necessary to ensure adequate oxygenation. The careful titration of oxygen is essential, as excessive oxygen can lead to respiratory acidosis in patients with chronic carbon dioxide retention [21]. Bronchodilators are the cornerstone of pharmacological therapy during exacerbations. Inhaled short-acting beta-agonists, often combined with anticholinergics, help alleviate dyspnea and improve lung function. These medications work by relaxing the bronchial muscles, thereby enhancing airflow and reducing the work of breathing. Alongside bronchodilators, systemic oral or intravenous corticosteroids are recommended to combat airway inflammation. Oral corticosteroids are preferred when gastrointestinal access is intact for hospitalized patients, as they can help reduce inflammation and improve respiratory function [22]. Antibiotics are indicated in cases where patients exhibit increased sputum purulence, as bacterial infections can exacerbate COPD symptoms. The choice of antibiotics should be guided by local resistance patterns, with common pathogens including *Streptococcus pneumoniae*, *Haemophilus influenzae*, and *Moraxella catarrhalis*. Timely initiation of antibiotics can help shorten the duration of exacerbations and reduce the risk of complications [23]. Noninvasive ventilation (NIV) has emerged as an effective strategy for managing acute or acute-on-chronic hypercapnic respiratory failure in COPD patients. NIV can improve respiratory acidosis, decrease respiratory rates, and alleviate breathlessness. Research has shown that it reduces the need for intubation, lowers mortality rates, and shortens hospital stays, making it a valuable tool in managing severe exacerbations [24]. In addition to pharmacological interventions, supportive measures are crucial in managing COPD exacerbations. Continuous monitoring of oxygen status, appropriate antibiotic administration, and managing comorbidities are essential components of care. Furthermore, initiating pulmonary rehabilitation within three weeks after hospital discharge can significantly enhance exercise capacity and improve the overall quality of life for patients recovering from exacerbations [25].

Postoperative Respiratory Support

HFNC oxygen therapy has several important clinical applications in critical care settings. HFNC is effective in treating patients with acute hypoxemic respiratory failure from various etiologies, including pneumonia and exacerbations of COPD. Studies show that HFNC can reduce breathing frequency and improve oxygen saturation compared to traditional oxygen therapy [26]. In addition, HFNC is increasingly used to prevent reintubation in patients who have undergone extubation, helping maintain adequate oxygenation and comfort during the critical period following extubation. HFNC can also be useful in avoiding oxygen desaturation during intubation in surgical patients and less severely ill critically ill patients. Available data indicate that HFNC may also facilitate weaning from mechanical ventilation. Furthermore, HFNC may benefit patients with do-not-intubate (DNI) orders, providing comfort and adequate oxygenation without invasive ventilation [26]. While HFNC has shown promise in these applications, further research is still needed to establish clear guidelines on when to initiate HFNC and which patient populations will benefit most. Strict monitoring is necessary, as delays in intubation due to neglected monitoring during HFNC use may result in poor patient outcomes [27]. Postoperative respiratory support is critical in managing patients after surgery, particularly those at risk of complications such as respiratory failure, pneumonia, and re-intubation. Various non-invasive respiratory support strategies have been investigated to enhance patient outcomes in the postoperative period [28]. HFNC provides heated and humidified oxygen at high flow rates, which can improve oxygenation and reduce the work of breathing. It has shown efficacy in preventing respiratory failure and re-intubation in postoperative patients. Studies indicate that HFNC can reduce the intubation rate and ICU-acquired infections, particularly in high-risk surgical populations [28].

COVID-19 and Other Infectious Diseases

HFNC oxygen therapy has been crucial in managing patients with COVID-19, especially those experiencing acute hypoxemic respiratory failure. HFNC provides a high fraction of humidified oxygen, which enhances ventilatory efficiency and patient comfort [29]. Studies have shown that HFNC can significantly reduce the need for IMV, with reports indicating that approximately 71% of patients treated with HFNC avoided intubation altogether. This non-invasive approach has been particularly valuable in critical care settings where resource demand has surged during the pandemic [29]. HFNC is often used as a first-line therapy for COVID-19 pneumonia, especially for patients with moderate to severe respiratory distress who have normal lung compliance and primarily require oxygen enrichment rather than invasive support. This therapy has also aided in better resource management in healthcare facilities, enabling treatment in non-ICU settings and optimizing care during periods of high patient volume. In addition, HFNC has been beneficial for post-extubation support, maintaining adequate oxygenation and comfort for patients recovering from invasive ventilation [30]. The outcomes associated with HFNC in the context of COVID-19 have been promising. A systematic review and meta-analysis demonstrated that HFNC significantly reduced the risk of intubation compared to conventional oxygen therapy (COT), with a risk ratio of 0.89, indicating a statistically significant reduction in the need for invasive support. HFNC therapy has also been linked to shorter hospital stays, which is advantageous for healthcare systems managing high patient volumes during surges in COVID-19 cases. However, while HFNC effectively reduces the need for intubation, its impact on mortality rates remains uncertain. The same meta-analysis found no significant reduction in mortality, suggesting that while HFNC effectively prevents intubation, it does not necessarily lead to lower mortality rates [31]. Despite its benefits, HFNC use has raised concerns about bioaerosol dispersion and the potential for increased infection transmission to healthcare workers. However, studies have indicated that HFNC can be safely used without widespread transmission risks, leading to its broader acceptance as a treatment modality during the pandemic [32].

Pediatric and Neonatal Applications

HFNC oxygen therapy has emerged as a valuable tool in pediatric and neonatal care, offering a non-invasive method for respiratory support. Its applications are broad, particularly in managing respiratory distress in infants and children. HFNC is frequently utilized in pediatric patients experiencing acute respiratory failure, with notable efficacy in moderate to severe bronchiolitis cases. Research suggests that HFNC reduces treatment failure rates compared to COT. Children generally well-tolerated it, making it a preferred option in emergency and inpatient settings. In the pediatric intensive care unit (PICU), HFNC proves effective as a post-extubation support system. It helps maintain adequate oxygenation and lowers the risk of reintubation in children who have recently been extubated, especially following surgical procedures or severe respiratory illnesses [33]. HFNC has also been evaluated for managing acute asthma exacerbations and other respiratory conditions. Although the evidence is still emerging, some studies suggest that HFNC may offer benefits comparable to CPAP while being better tolerated by younger patients. Initially introduced for neonates, HFNC has effectively prevented reintubation and provided initial noninvasive respiratory support in preterm infants. It has proven useful in managing apnea and improving oxygenation without the discomfort of invasive ventilation methods [34]. Despite its growing use, the literature on HFNC in pediatric and neonatal care is still developing. More randomized controlled trials (RCTs) are needed to establish clear guidelines regarding their use, including optimal flow rates, indications, and contraindications. Future research should expand HFNC applications beyond bronchiolitis and assess its cost-effectiveness in various clinical scenarios. Overall, HFNC therapy represents a significant advancement in pediatric respiratory care, offering a versatile and effective option for managing various respiratory conditions in infants and children [35].

Evidence-based efficacy

Clinical Trials and Studies

Recent clinical trials and meta-analyses have highlighted the efficacy of HFNO therapy in critical care settings. One landmark study is the FLORALI, a multicenter, randomized, open-label trial involving 310 adult ICU patients. This study found no significant difference in 28-day intubation rates among patients receiving HFNO (38%), NIV (50%), and COT (47%). However, HFNO was associated with lower in-ICU and 90-day mortality rates than NIV and COT, indicating potential survival benefits [36]. Another significant study, the HIGH trial, evaluated HFNO in 776 immunocompromised ICU patients. There were no notable differences in 28- or 90-day mortality rates between HFNO and COT. The incidence of IMV was similar between the HFNO (38.7%) and COT (43.8%) groups, suggesting that while HFNO may not drastically reduce intubation rates in this population, it remains a viable respiratory support option [37]. In addition, a controlled trial by Andino et al., involving 46 ICU patients, demonstrated that HFNO resulted in significantly fewer intubations (33%) than COT (63%). Patients receiving HFNO showed improved arterial blood gas parameters, including PaO_2_/FiO_2_ and PaCO_2_ levels, although there were no significant differences in mortality or comfort levels. Furthermore, Coudroy et al.'s study, which included 300 immunocompromised ICU patients, found no significant differences in 28-day mortality rates between HFNO alone (36%) and a strategy alternating NIV with HFNO (35%), underscoring the need for additional research [38]. These findings collectively suggest that while HFNO effectively improves oxygenation and reduces the need for intubation, its impact on mortality remains uncertain across various patient populations. Comparatively, HFNO has been shown to provide superior oxygenation compared to conventional methods, enhancing arterial oxygen levels and reducing the work of breathing, especially in patients with hypoxemic respiratory failure [39]. Despite its association with reduced intubation rates in some studies, its efficacy relative to NIV varies. For instance, the FLORALI trial, while not showing a significant reduction in intubation rates compared to NIV, indicated a trend toward better mortality outcomes with HFNO, suggesting possible advantages in certain clinical scenarios [40]. Patient comfort is another area where HFNO often excels. It is generally better tolerated than NIV, offering patients greater freedom of movement and comfort, which is crucial in the high-stress ICU environment. In addition, HFNO's portability compared to NIV enhances its flexibility and usability in different hospital settings, improving overall patient care [40].

Patient Outcomes

HFNO therapy has demonstrated notable benefits in critical care settings, particularly in improving patient outcomes related to survival rates, intubation rates, and length of ICU stay. HFNO has been associated with enhanced survival rates in patients with acute hypoxemic respiratory failure. Systematic reviews and meta-analyses of RCTs consistently show that HFNO therapy significantly reduces mortality compared to standard oxygen therapy, especially in conditions like pneumonia and ARDS. Notably, in patients with COVID-19-related respiratory failure, HFNO has shown promising survival benefits, with some studies reporting survival rates exceeding 80% among HFNO recipients, compared to lower rates observed with conventional oxygen therapy [26]. One of HFNO's key advantages is its effectiveness in reducing the need for IMV. Research consistently shows that HFNO can lower intubation rates by approximately 30-50% in patients with acute hypoxemic respiratory failure. For instance, a study comparing HFNO to standard oxygen therapy found a significant reduction in intubation rates, with HFNO recipients experiencing rates around 15%, compared to 30% in those receiving standard therapy. HFNO has also been successfully used as a preventive measure in high-risk populations, such as those with COPD or patients experiencing post-extubation respiratory failure, further contributing to decreased intubation rates [38]. In addition, HFNO is associated with a reduction in the ICU length of stay. Studies indicate that patients on HFNO typically have shorter ICU stays than those receiving standard oxygen therapy or NIV. For example, the median ICU stay for HFNO patients averages around five days, compared to seven to 10 days for those on alternative respiratory support methods. This reduction benefits patients by promoting faster recovery and improves healthcare resource management by optimizing ICU capacity [41].

Patient Comfort and Tolerance

HFNO therapy significantly improves patients' comfort and tolerance, especially in critical care settings. Delivered through a comfortable nasal cannula, HFNO is generally better tolerated than other NIV methods, such as non-invasive positive pressure ventilation (NIPPV). This enhanced comfort is due to several factors: HFNO is less invasive than mechanical ventilation, allows easier interaction with caregivers, and reduces anxiety associated with more cumbersome devices [2]. In addition, the therapy provides heated and humidified oxygen, which helps prevent dryness and irritation in the airways, further contributing to overall comfort. HFNO also reduces the work of breathing and inspiratory effort, alleviating discomfort associated with respiratory distress. Studies have shown that patients on HFNO experience a decrease in respiratory rate and an increase in oxygenation levels, enhancing their overall comfort during treatment [2]. Research indicates that patients find HFNO significantly more tolerable than traditional oxygen therapies and NIPPV. For instance, a study demonstrated that HFNO was better tolerated, leading to improved oxygenation and reduced tachypnea compared to standard oxygen therapy. Patients reported a higher comfort level with HFNO, which facilitated normal activities like eating, compared to conventional oxygen methods. The comfort and tolerance associated with HFNO have important clinical implications, such as reducing the need for IMV and serving as an effective bridge between different forms of respiratory support. This allows patients to maintain adequate oxygenation and comfort during therapy transitions [12].

Safety and complications

Common Side Effects

HFNC oxygen therapy is generally considered safe and well-tolerated; however, like any medical intervention, it can be associated with certain side effects and complications. Understanding these potential issues is crucial for healthcare providers to ensure optimal patient care. One of the most common side effects of HFNC is skin breakdown. Prolonged use can lead to localized skin damage, particularly in the nasal philtrum and areas beneath the straps of the cannula. While this risk exists, it is notably lower than complications associated with NIV or CPAP therapies. Healthcare providers should regularly assess the skin condition of patients on HFNC to prevent and address any issues promptly [42]. Patients may also experience temperature discomfort when HFNC is first initiated. This discomfort typically arises from the flow rate or temperature of the heated and humidified oxygen. However, most patients acclimate to the therapy within the first few minutes, and this sensation generally resolves quickly. Clinicians should reassure patients and adjust the settings as necessary to enhance comfort [42]. Another significant concern with HFNC therapy is the potential for air-leak syndromes, such as pneumothorax, pneumomediastinum, or subcutaneous emphysema. Although these complications are rare, they can occur, and clinicians must remain vigilant. HFNC can sometimes mask clinical deterioration, potentially delaying necessary interventions like intubation. Observational studies suggest that patients who require intubation after prolonged HFNC treatment may have a higher mortality risk compared to those who are intubated earlier. Therefore, if a patient shows no improvement with HFNC, timely escalation to invasive ventilation is critical [43]. In addition, patients receiving HFNC may be at risk for ventilator-associated pneumonia (VAP), particularly in settings such as the ICU. A study involving COVID-19 patients reported that VAP was the most common adverse event, occurring in 58% of those treated with HFNC. This underscores the importance of maintaining strict infection control measures and monitoring for signs of respiratory infection in patients undergoing HFNC therapy [31].

Potential Risks and Adverse Events

HFNC therapy is safe, but clinicians should be aware of potential risks and adverse events. Case reports have documented air leak syndromes such as pneumothorax, pneumomediastinum, or subcutaneous emphysema associated with HFNC use. Clinicians must exercise caution, as HFNC can sometimes mask clinical deterioration and delay necessary interventions like intubation. For instance, a study found that new pneumothoraces occurred in 1% of children treated with HFNC in the pediatric ICU, highlighting that while these complications are rare, they can be serious [44]. Observational studies suggest that patients who require intubation after prolonged HFNC treatment may have a higher mortality risk compared to those who are intubated earlier. This underscores the importance of close monitoring and avoiding delays in intubation if the patient’s condition is deteriorating. In addition, clinically significant epistaxis was reported in 0.6% of children treated with HFNC in one study, with high flow rates potentially causing nasal irritation, and the safety of enteral feeding while on HFNC remains uncertain. The positive pressure from HFNC might disrupt the suck-swallow-breathe reflex and increase the risk of aspiration. More research is needed to establish safe feeding practices during HFNC therapy [38]. Some patients with neuromuscular diseases may be intolerant of HFNC due to discomfort, agitation, or uncooperativeness. Close monitoring is essential to ensure patient tolerance of the therapy. While HFNC is generally safe, potential risks include air leaks, delayed intubation, epistaxis, feeding intolerance, and patient intolerance. Careful patient selection, close monitoring, and a low threshold for intubation if deterioration occurs are crucial to maximize safety. Further research is needed to understand HFNC safety, particularly in non-ICU settings [45].

Guidelines for Monitoring and Management

HFNC therapy has become essential for managing acute respiratory failure, particularly in critical care settings. To ensure patient safety and optimize therapeutic outcomes, adherence to established guidelines for monitoring and management is crucial. Continuous monitoring is fundamental to effective HFNC therapy. Pulse oximetry should be used to continuously assess oxygen saturation (SpO_2_), especially during the initial hours of treatment. This enables healthcare providers to make timely adjustments to flow rates and the fraction of inspired oxygen (FiO_2_) as needed. Alongside pulse oximetry, regular clinical assessments are essential. Clinicians should monitor respiratory rate, work of breathing, and overall patient comfort, watching for signs of respiratory distress or failure. In some cases, particularly with severe respiratory failure or inadequate response to initial HFNC treatment, arterial blood gas (ABG) analysis may be required [46]. When starting HFNC, selecting appropriate flow rates, typically 30 to 60 L/min, is important based on the individual patient's needs. FiO_2_ should be adjusted to maintain target SpO_2_ levels between 92% and 96%. As patients stabilize, healthcare providers should implement a weaning protocol, evaluating the readiness to reduce flow rates or FiO_2_ at least once per shift. This gradual approach helps prevent premature discontinuation of HFNC support [47]. Special considerations are necessary when using HFNC. Patient selection is critical; HFNC is particularly beneficial for those with acute hypoxemic respiratory failure, but caution is needed for patients with anatomical abnormalities or a high risk of aspiration. Appropriate infection control measures must be implemented because HFNC is classified as an aerosol-generating procedure (AGP). This includes using personal protective equipment (PPE) and, when possible, conducting therapy in a negative pressure room, especially for patients with infectious respiratory illnesses [1].

Advantages and limitations

Benefits of HFNC

HFNC oxygen therapy offers several notable advantages for managing respiratory failure, particularly in critical care settings. One of the primary benefits is its non-invasive nature. Unlike invasive procedures such as intubation, HFNC provides a safer alternative with a significantly lower risk of complications. This is especially beneficial for patients at high risk of complications or those with DNI orders. In addition, HFNC avoids the need for an artificial airway, reducing the risk of VAP and other nosocomial infections and making it a safer choice for managing respiratory distress [2]. HFNC also enhances patient comfort compared to traditional oxygen delivery methods. Many individuals find HFNC more tolerable than face masks or NIV. The lightweight nasal cannula allows for greater mobility and reduces feelings of claustrophobia, which can improve patient adherence to the therapy. Moreover, HFNC delivers heated and humidified oxygen, which helps maintain mucosal integrity and comfort. This is particularly important for patients with dry or irritated airways, as it alleviates the discomfort commonly associated with conventional oxygen therapy. Patients using HFNC often report significantly reduced dyspnea or breathlessness, improving comfort and quality of life during treatment [34]. In addition, HFNC offers flexibility and ease of use. It can be applied in various clinical scenarios, including ARDS, pneumonia, exacerbations of COPD, and post-extubation support. This versatility makes HFNC suitable for intensive care units (ICU) and non-ICU settings. The equipment for HFNC is relatively straightforward to set up and operate, facilitating its use by nursing staff and allowing for rapid initiation of therapy in emergencies. Furthermore, HFNC permits precise adjustments in flow rates (up to 60 L/min) and the fraction of inspired oxygen (FiO_2_), enabling healthcare providers to tailor the therapy to patients' needs. This adaptability is crucial for managing varying degrees of hypoxemia and respiratory distress [1].

Limitations and Challenges

While HFNC oxygen therapy has demonstrated promising outcomes in critical care settings, it has its limitations and challenges. Two major areas that warrant further attention are equipment and resource constraints and clinical indications and contraindications. A significant limitation is the cost associated with HFNC systems, which can be higher than traditional oxygen delivery methods. This financial burden may restrict HFNC availability in resource-constrained settings, such as developing countries or smaller healthcare facilities. Moreover, the specialized equipment required for HFNC may not be readily accessible in all locations, potentially hindering its broader adoption [48]. Proper implementation of HFNC also requires adequate training for healthcare professionals and regular equipment maintenance to ensure optimal performance and patient safety. Inadequate training and maintenance protocols can compromise the effectiveness of HFNC therapy [27]. Another critical consideration is patient selection. Identifying appropriate candidates for HFNC therapy is essential, as inappropriate use may delay necessary intubation and worsen patient outcomes. Standardized guidelines are needed to define patient selection criteria and ensure HFNC is used in suitable clinical scenarios [34]. HFNC may not be appropriate for all patients. Contraindications include upper airway obstruction, severe hemodynamic instability, and the inability to protect the airway. Healthcare providers must carefully evaluate each patient’s condition before initiating HFNC therapy to avoid potential complications [48]. Although HFNC can sometimes help avoid intubation, it is crucial to recognize when a patient's condition is deteriorating and escalate care as needed. Delayed intubation due to overreliance on HFNC may lead to adverse outcomes, highlighting the importance of close monitoring and frequent adjustments of flow rates and FiO2 to ensure optimal oxygenation and prevent complications such as nasal mucosa drying or gastric distension [1]. Healthcare systems must invest in the necessary equipment and training to address these limitations and challenges. At the same time, researchers and clinicians collaborate to establish clear guidelines for patient selection, monitoring, and escalation of care. Ongoing research is essential to refine HFNC use in critical care settings, maximizing its benefits while minimizing potential risks [49].

Future directions

Emerging Research and Innovations

Emerging research and innovations in HFNC therapy are expanding its applications and deepening our understanding of its benefits and limitations across various clinical settings. One prominent area of exploration is using HFNC for chronic respiratory diseases, such as COPD. Recent studies indicate that HFNC may reduce exacerbation rates and enhance quality of life when utilized as a domiciliary treatment. This potential for long-term management underscores the need for further research into its effectiveness in outpatient settings [50]. HFNC is also gaining recognition in palliative care for managing dyspnea in patients with life-limiting illnesses. Its non-invasive nature and capacity to deliver high oxygen levels make it an attractive option for symptom relief. However, further research is needed to compare HFNC's efficacy against traditional therapies, such as opioids and NIV, to better define its role in this context. In the postoperative realm, there is growing interest in using HFNC for patients at risk of respiratory failure. Investigations are needed to assess its effectiveness in preventing postoperative complications compared to standard oxygen therapies, potentially leading to enhanced recovery protocols [51]. Future research should focus on identifying specific patient populations that benefit most from HFNC, including those with congestive heart failure, septic shock, or moderate to severe hypoxemia. Understanding these demographics will aid in tailoring HFNC protocols and optimizing patient outcomes. Comparative studies are also essential to evaluate HFNC against other non-invasive methods, particularly NIV, to establish clear guidelines for their respective uses [27]. Technological innovations are poised to advance HFNC therapy further. Ongoing improvements in device technology, such as enhanced humidification and heated delivery systems, may increase patient comfort and improve clinical outcomes. Telemonitoring with HFNC therapy could enhance patient compliance and outcomes, particularly in home settings. This approach could facilitate better management of chronic respiratory conditions and help reduce hospital readmissions [26].

Potential for Integration With Other Therapies

Integrating HFNC therapy with other treatment modalities holds considerable promise for improving patient outcomes in critical care settings. One notable area of interest is the combined use of HFNC with NIV [52]. Although HFNC is recognized for its simplicity and superior patient tolerance compared to NIV, exploring their sequential or simultaneous use could enhance oxygenation and patient comfort. Research should focus on comparative effectiveness studies to identify which patient groups, such as those with severe hypoxemia from COPD exacerbations or cardiogenic pulmonary edema, might benefit most from this combined approach [52]. Another crucial area for integration is prehospital and transport settings. HFNC has demonstrated potential in non-critical care transport scenarios, particularly for pediatric and neonatal patients. Future studies should evaluate the safety and efficacy of HFNC compared to traditional oxygen delivery methods during patient transport, especially in emergencies. Developing guidelines for HFNC use in prehospital settings is essential to ensure proper monitoring and supervision, thereby mitigating potential adverse outcomes [53]. Integrating HFNC with other therapeutic strategies in the perioperative setting presents significant opportunities. For example, using HFNC for preoxygenation in patients at risk of hypoxemia during anesthesia induction could reduce intraoperative complications. In addition, examining HFNC's role in postoperative recovery, especially for high-risk surgical patients, may help prevent respiratory complications and improve recovery times [11].

Advances in Technology and Equipment

HFNC oxygen therapy has seen remarkable technological advancements in recent years, significantly enhancing its efficacy and practicality in critical care settings. A key development is the integration of HFNC modes into contemporary ventilators. These advanced devices allow clinicians to switch seamlessly between HFNC, NIV, and invasive ventilation modes. This integration simplifies patient management and reduces the need for multiple equipment, facilitating more efficient delivery of respiratory support [54]. Another notable innovation is the implementation of closed-loop ventilation systems. These systems leverage sophisticated algorithms and artificial intelligence to automatically adjust HFNC settings based on real-time monitoring of a patient’s breathing patterns. By reducing the need for manual adjustments, closed-loop technology offers personalized ventilation support, potentially improving patient outcomes and comfort [55]. Ventilator-integrated tomography (VIT) represents a groundbreaking advancement in lung function assessment. This technology enables continuous imaging without requiring X-rays, allowing for real-time monitoring of respiratory status. When combined with HFNC measurement and control, VIT provides clinicians with valuable insights for making more informed therapeutic decisions and enhancing the management of respiratory conditions [56]. Remote patient monitoring is another significant enhancement in HFNC technology. Newer devices equipped with wireless connectivity allow healthcare providers to monitor patients and adjust settings in real time, even from a distance. This capability is particularly advantageous for managing patients in non-ICU settings or during transport, where timely interventions are crucial [57].

Recommendations for Future Studies and Clinical Trials

Future studies and clinical trials on HFNC therapy should focus on several key areas to enhance understanding and optimize its use in critical care settings. Research should aim to identify specific patient groups that may benefit most from HFNC, such as those with congestive heart failure, COPD, and septic shock. Understanding HFNC's effectiveness in these populations will help refine treatment protocols [26]. Comparative studies are needed to evaluate HFNC against other NIV strategies, particularly NIPPV. This includes assessing outcomes in various clinical scenarios, such as postoperative settings and acute respiratory failure. In addition, future research should focus on establishing clear protocols for HFNC initiation, escalation, and weaning. This involves determining optimal settings for flow rates and FiO_2_ and identifying early signs of HFNC failure to prevent delays in more invasive interventions [58]. Long-term outcome studies are necessary to evaluate the cost-effectiveness of HFNC across different healthcare systems. This will provide insights into its economic viability and impact on healthcare resources. Research should also explore the potential synergistic benefits of combining HFNC with other therapies, such as NIPPV, and how these combinations can improve patient outcomes in various clinical contexts [59]. Furthermore, studies should assess HFNC's effectiveness in different healthcare settings, including ICUs, wards, and emergency departments. Evaluating its use in low-income countries, where the etiologies of respiratory failure may differ, is also crucial. More research is needed in pediatric populations to explore HFNC's effectiveness, especially in children with severe respiratory illnesses. Studies should focus on identifying optimal initiation and monitoring practices to minimize the risk of failure. In addition, trials are needed to refine postoperative care strategies to investigate HFNC's role in postoperative patients at risk of respiratory failure [27].

## Conclusions

HFNC therapy has emerged as a transformative tool in critical care, offering significant advantages over traditional oxygen delivery methods. By delivering high-flow, humidified, and heated oxygen, HFNC enhances patient comfort, improves respiratory function, and reduces the need for invasive interventions. The evolution of HFNC technology and its application in managing acute and chronic respiratory conditions underscore its importance in modern critical care. Current evidence supports its efficacy in improving patient outcomes, such as reducing intubation rates and shortening ICU stays. However, while HFNC therapy demonstrates substantial benefits, ongoing research is crucial to address its limitations and optimize its use. Future studies should focus on refining protocols, expanding indications, and integrating HFNC with other therapeutic modalities to further advance respiratory care. Ultimately, HFNC therapy represents a significant advancement in respiratory support, potentially enhancing patient outcomes and redefining critical care practices.
